# Performance of Interferon-Gamma Release Assays in the Diagnosis of Nontuberculous Mycobacterial Diseases—A Retrospective Survey From 2011 to 2019

**DOI:** 10.3389/fcimb.2020.571230

**Published:** 2021-02-18

**Authors:** Chi Yang, Xuejiao Luo, Lin Fan, Wei Sha, Heping Xiao, Haiyan Cui

**Affiliations:** Shanghai Clinical Research Center for Tuberculosis, Shanghai Pulmonary Hospital, Tongji University School of Medicine, Shanghai, China

**Keywords:** NTM disease, diagnose performance, IGRAs, QFT-G, T-SPOT.TB.

## Abstract

There is an urgent need for precise diagnosis to distinguish nontuberculous mycobacterial (NTM) diseases from pulmonary tuberculosis (PTB) and other respiratory diseases. The aim of this study is to evaluate the diagnostic performance of Interferon-gamma (IFN-γ) release assays (IGRAs), including antigen-specific peripheral blood-based quantitative T cell assay (T-SPOT.TB) and QuantiFERON-TB-Gold-Test (QFT-G), in differentiating NTM infections (*N* = 1,407) from culture-confirmed PTB (*N* = 1,828) and other respiratory diseases (*N* = 2,652). At specie level, 2.56%, 10.73%, and 16.49% of NTM-infected patients were infected by *Mycobacterium kansasii*, *M. abscessus*, and with *M. avmm*-*intracellulare* complex (MAC), respectively. Valid analyses of T-SPOT.TB (ESAT-6, CFP-10) and QFT-G were available for 37.03% and 85.79% in NTM-infected patients, including zero and 100% (36/36) of *M. kansasii* infection, 21.85% (33/151) and 92.05% (139/151) of *M. abscessus* infection, and 17.67% (41/232) and 91.24% (211/232) of MAC infection. Based on means comparisons and further ROC analysis, T-SPOT.TB and QFT-G performed moderate accuracy when discriminating NTM from PTB at modified cut-off values (ESAT-6 < 4 SFCs, CFP-10 < 3 SFCs, and QFT-G < 0.667 IU/ml), with corresponding AUC values of 0.7560, 0.7699, and 0.856. At species level of NTM, QFT-G effectively distinguished between MAC (AUC=0.8778), *M. kansasii* (AUC=0.8834) or *M. abscessus* (AUC=0.8783) than T-SPOT.TB. No significant differences in discriminatory power of these three IGRA tools were observed when differentiating NTM and Controls. Our results demonstrated that T-SPOT.TB and QFT-G were both efficient methods for differentiating NTM disease from PTB, and QFT-G possessed sufficient discriminatory power to distinguish infections by different NTM species.

## Introduction

*Mycobacteria* are a group of extremely diverse and ubiquitous microorganisms and inhabit nearly every environmental niches (von Reyn et al., 1993; Falkinham, 2002; Johansen et al., 2020), consisting of two major categories: tuberculosis (TB) - causing mycobacteria (MTB) and non-tuberculous mycobacteria (NTM) (Runyon, 1959; Wolinsky, 1992). Partial NTM (*M. avium Complex (MAC), M. kansasii, M. abscessus, M. chelonae, M. fortuitum, M. genavense, M. gordonae, M. haemophilum, M. immunogenum, M. malmoense, M. marinum, M. mucogenicum, M. nonchromogenicum, M. scrofulaceum, M. simiae, M. smegmatis, M. szulgai, M. terrae complex, M. ulcerans, M. xenopi*) are opportunistic pathogens to humans and are the cause of most common lung diseases in clinical with rapidly increasing prevalence worldwide, especially in immuno-compromised patients (Marras et al., 2007; Billinger et al., 2009; Prevots et al., 2010; Thomson et al., 2010; Winthrop et al., 2010; Tsai et al., 2011). The microscopic examination of sputum for acid-fast bacilli (AFB) is a diagnostic standard of pulmonary tuberculosis (PTB). However, the AFB smear-positive are also present in NTM infection. The recovery rate of NTM in AFB positive patients was already considerably high with geographical variation, for instance, 48.5% in the United States (Wright et al., 1998), 43.2% in Australia (Anargyros et al., 1990), 21.1% in Spain (Coll et al., 2003) and 9.1% in Korea (Jeon et al., 2005; Glassroth, 2008; Ryoo et al., 2008). Thus, early clinical identification of NTM infection and PTB would be helpful in patients with AFB smear-positive sputum, as well as for NTM infection and other respiratory diseases with AFB smear-negative sputum (Griffith et al., 2007). However, due to similar clinical symptoms of these lung disease, traditional diagnostic methods, including tuberculin skin test (TST or Mantoux) and chest-X-ray (CXR) are considered unreliable in the diagnosis of MTB. Several molecular techniques (PCR restriction analysis, Anyplex MTB/NTM detection assay, GenoType Mycobacteria Direct test) had been developed for early NTM detection and already been commercially available. However, these tools were regarded to be costly, less sensitive than conventional acid-fast bacilli (AFB) and therefore not recommended in routine clinical practice by British Thoracic Society guidelines at the present time (Haworth et al., 2017). Therefore, there is an urgent need of an early, fast diagnostic technology to distinguish NTM infection from PTB, and from other respiratory diseases (Huebner et al., 1993; Kim et al., 2014).

Interferon-gamma (IFN-γ) release assays (IGRAs), including T-SPOT.TB and QFT-G, display a higher sensitivity compared to the TST for specific detection of latent TB, pulmonary TB or extrapulmonary TB, based on the T-cell mediated IFN-γ release induced by specific *M. tuberculosis* antigens, including ESAT-6, CFP-10 and TB7.7. These specific peptide antigens are usually located in the region of difference (RD1) of MTB genome, and RD1 usually exists in various species of mycobacteria belonging to the *M. tuberculosis* complex (*M. tuberculosis*, *M. bovis*, *M. africanum*, *M. canettii*, *M. caprae*, *M. orygis*, *M. microti*, *M. pinnipedii*, and *M. mungi*) (van Ingen et al., 2012), while only a few species of NTM (*M. kansasii*, *M. gastri*, *M. marinum*, *M. szulgai*, and *M. riyadhens*e) share the similar RD1 areas (Harboe et al., 1996; Mahairas et al., 1996; van Ingen et al., 2009). Therefore, IGRAs present high sensitivity for discriminating NTM and MTB.

The aim of this study is to evaluate the efficiency of three different IGRAs (ESAT-6, CFP-10 and QFT-G) for diagnosing NTM infection from PTB and other respiratory diseases.

## Material and Methods

### Patient Population and Ethics Statement

This retrospective study collected clinical data from the Shanghai Pulmonary Hospital, Tongji University School of Medicine (Shanghai, P.R. China) between October 2011 to July 2019. In total, 1,407 consecutive patients diagnosed with NTM infection by culture for mycobacteria were enrolled. 1,828 patients with culture-confirmed pulmonary tuberculosis (PTB) and 2,652 patients with respiratory diseases (pneumonia, pulmonary malignancy, bronchiectasis *etc.*) excluding those infected with NTM or PTB, were enrolled as controls. These respiratory diseases cases without positive results from NTM and mycobacterial culture were mainly diagnosed by etiology, clinical symptoms, imaging findings or pathological examination. The Institutional Review Board of Shanghai Pulmonary Hospital affiliated with Tongji University approved the study and waived the need for informed consent since no patients were at risk. All clinical records were anonymized and de-identified prior to analysis.

### Classification and Diagnosis

NTM diseases were diagnosed with modified guidelines of the American Thoracic Society (ATS) and the Infectious Disease Society of America (IDSA) 2007 criteria (Griffith et al., 2007; Andrejak et al., 2010). Patients with positive culture for NTM from extra pulmonary sites (skin, lymph nodes *etc.*) were also included in line with Freeman et al. (2007). The NTM-infected patients with a previous history of TB disease or MTB isolations from clinical specimens were excluded. Patients were excluded due to discordant IGRAs results, or results not within a 6 month period before or after the positive NTM culture. PTB was diagnosed by sputum culture according to the World Health Organization guidelines (WHO, 2010).

1,407 NTM-infected patients were included including 36 identified as *M. kansasii* infection, 151 as *M. abscessus* infection and 232 as MAC infection. There were 2,652 control patients without NTM and PTB, including 941 (35%) with pneumonia, 599 (23%) with a pulmonary malignancy and 358 (13%) with bronchiectasis. All the participants (*n* = 198) had negative results on serological tests for human immunodeficiency virus (HIV). The demographic and clinical characteristics of all participants are shown in **Table 1**.

**Table 1:** Demographic and clinical characteristics of patients with participants.

index	*N*TM	Three major *N*TMs			PTB	Co*n*trols
	*N* = 1,407	*M.kansasii N* =36	*M. abscessus N* =151	MAC *N* =232	*N* = 1,828	*N* = 2,652
Age, years	60 ± 15	50 ± 14	59 ± 13	60 ± 14	43 ± 18	56 ± 16
Sex, male	610(43)	25(69)	51(34)	91(39)	1,230(67)	1,593(60)
Concomitant diseases (%)						
Diabetes mellitus	79(6)	0(0)	3(2)	11(5)	242(13)	258(10)
Malignancy	55(4)	0(0)	4(3)	6(3)	66(4)	655(25)
Rheumatic disease	39(3)	1(3)	4(3)	8(3)	19(1)	43(2)
Coronary heart disease	29(2)	0(0)	0(0)	4(2)	22(1)	82(3)
Hypertension	154(11)	2(6)	13(9)	28(1)	141(8)	496(19)

Data presented as mean ± SD or n of patients (%). N represented for enrolled patient number.

### Laboratory Tests and Examination

All bacterial cultures were assessed using the BD BACTEC™ MGIT™ automated mycobacterial detection system (Becton, USA) and all mycobacterial cultures were evaluated using the BD BACTEC™ MGIT™ automated mycobacterial detection system (Becton, Dickinson and Company, Franklin Lakes, NJ, USA). Subsequently, partial species (MAC, *M. kansasii M. abscessus M. gastri*, *M. marinum*, M. szulgai *etc.*) of NTM were identified by 16S rRNA gene sequencing as described previously (Hall et al., 2003). The T-SPOT^®^.TB assays were conducted following manufacturer’s instructions (Oxford, UK). Briefly, all blood samples were collected immediately prior to the tests in order to reduce potential interferences. Peripheral blood mononuclear cells (PBMCs) were isolated from a whole blood sample using Ficoll-Hypaque gradient centrifugation at 400 × g for 30 min at 20°C. Then, the PBMCs were incubated with antigens to stimulate INF-γ secretion by the T cells, and seeded on precoated IFN-γ ELISpot plates followed by incubation with a medium without an antigen (negative control), or a medium containing peptide antigens from ESAT-6 (panel A) or peptide antigens from CFP-10 (panel B), or a medium containing phytohemagglutinin (positive control) in a 5% CO_2_ atmosphere at 37°C for 20 h (Wang et al., 2010; Wang et al., 2012). The spot-forming cells were counted by an ELISPOT plate reader (AID-GmbH, Straßberg, Germany). Quantitative results for the T-SPOT.TB test are interpreted by subtracting the spot count in the negative controls well from the spot count in each of the Panels, and this number must be at least two-times greater than the spot-forming cells (SFCs) number from the negative wells (Bouwman et al., 2012). All tests were performed before anti-TB medication. The QuantiFERON-TB-Gold-Test (Cellestis Ltd., Carnegie, Victoria, Australia) was also performed following the manufacturer’s recommendations. Briefly, aliquots of heparinized whole blood are incubated with the test antigens (ESAT-6, CFP-10, and TB 7.7 proteins) for 16–24 h; phytohemaglutinin is performed as the positive assay control, and saline as the negative control (nil tube). After incubation, the concentration of IFN-g in the plasma would be read by ELISA and the quantitative result of the test was reported as the IFN-γ level in the sample tube minus the baseline level (nil tube) (Mazurek et al., 2005).

### Statistical Analyses

All data were analyzed by using MedCalc^®^ version 9.0.1.1 (MedCalc, Belgium). The results by different tests were compared using χ2-test to assess the potential to discriminate each other. Then, the ROC curves were calculated between the groups with statistically significant difference. Areas under the ROC curve (AUC) are evaluated to assess the discriminatory powers of IGRA test. Generally, the AUC values are positive correlation to reliability and discrimination, in which higher than 0.9 indicates high accuracy, 0.7–0.9 indicates moderate accuracy, 0.5–0.7 indicates low accuracy, and less than 0.5 indicates no discrimination (Oh et al., 1993; Fischer et al., 2003). An AUC value greater than 0.7 on the validation can be considered as acceptable models for differentiation. The corresponding index, including optimal cut-off values, sensitivity, specificity, positive predictive value (PPV), negative predictive value (NPV) are calculated.

## Results

In total, valid results of T-SPOT.TB were available for 37.03% (521/1,407) in NTM-infected patients, 30.03% (549/1,828) in PTB patients and 48.57% (1,288/2,652) in controls. For QFT-G, valid results were available for 73.13% (1,029/1,407) in NTM-infected patients, 69.97% (1,279/1,828) in PTB patients and 51.43% (1,364/2,652) in controls. At the species level of NTM, the valid results of T-SPOT.TB were available for 21.85% (33/151) in patients infected with *M. abscessus* and 17.67% (41/232) with MAC. For QFT-G, valid results were available for 100% (36/36) of patients infected with *M. kansasii*, 92.05% (139/151) with *M. abscessus* and 90.95% (211/232) with MAC (**Table S1**). Quantitative results of different IGRAs were compared (**Table S2**). The *P* values indicated both T-SPOT.TB and QFT-G were effective when discriminating NTM form PTB (*P* < 0.001), while QFT-G showed lower performance (*P* = 0.1853) than T-SPOT.TB (*P* = 0.0000 for ESAT-6 and *P* = 0.0017 for CFP-10) when discriminating NTM from controls.

To further evaluate the diagnostic performance of IGRAs when discriminating NTM from PTB, the ROC analyses with statistical significances were conducted. As shown in **Table 2**, ESAT-6 (AUC: 0.7560), CFP-10 (AUC: 0.7699) and QFT-G (AUC: 0.8560) had moderate accuracy with AUC > 0.7 (**Figure S1**). Notably, based on sensitivity, specificity, PPV, NPV, and AUC, QFT-G showed better diagnostic performance than ESAT-6 and CFP-10. When discriminating NTM from Controls, ESAT-6 and CFP-10 displayed low accuracy with AUC < 0.7 (**Figure S2**, **Table 3**). At the species level of NTM, ESAT-6 and CFP-10 both had moderate accuracy with AUC > 0.7 when discriminating MAC or *M abscessus* from PTB. QFT-G also performed moderate accuracy with AUC > 0.7 when discriminating MAC, *M. kansasii* or *M. abscessus* from PTB, However, QFT-G established better performance either ESAT-6 or CFP-10 when discriminating MAC or *M. abscessus* from PTB (**Figure S3**, **Table 4**).

**Table 2:** Diagnostic performance of interferon-gamma release assays (IGRA) tools to discriminate nontuberculous mycobacterial (NTM) from pulmonary tuberculosis (PTB).

Index	QFT-G	ESAT-6	CFP-10
**Cut-off value to distinguish NTM**	<0.667 IU/ml	<4 SFCs	<3 SFCs
**Sensitivity (%)**	0.867	0.7942	0.7596
**Specificity (%)**	0.7483	0.6315	0.6756
**PPV (%)**	0.7349	0.6716	0.6897
**NPV (%)**	0.8750	0.7638	0.7475
**AUC**	0.8560	0.7560	0.7699
**95%CI**	0.840–0.8719	0.7268–0.7852	0.7415–0.7983
***P*-value**	0.0000	0.0000	0.0000

**Table 3:** Diagnostic performance of T-SPOT.TB to discriminate nontuberculous mycobacterial (NTM) from controls.

Index	ESAT-6	CFP-10
**Cut-off value**	>1 SFCs	>2 SFCs
**Sensitivity (%)**	0.4664	0.3244
**Specificity (%)**	0.6421	0.7539
**PPV (%)**	0.3452	0.3477
**NPV (%)**	0.7484	0.7339
**AUC (95% CI)** **95% CI** ***P*-value**	0.5537 0.5236–0.5838 P=0.0003	0.5458 0.5160–0.5756 P=0.002

**Table 4:** Diagnostic performance of interferon-gamma release assay (IGRA) tools to discriminate species of nontuberculous mycobacterial (NTM) from pulmonary tuberculosis (PTB).

	Index	QFT-G	ESAT-6	CFP-10
**MAC from PTB**	Cut-off value	<0.6670 IU/ml	<2 SFCs	<2 SFCs
	Sensitivity (%)	0.8671	0.8525	0.7996
	Specificity (%)	0.8389	0.6829	0.7805
	PPV (%)	0.4703	0.1672	0.2139
	NPV (%)	0.9745	0.9841	0.9812
	AUC (95%CI) 95% CI *P* value	0.8778 0.8479–0.9077 0.0000	0.8226 0.7583–0.8868 0.0000	0.8183 0.7536–0.8830 0.0000
***M. kansasii* from PTB**	Cut-off value	<0.8180 IU/ml	−	−
	Sensitivity (%)	0.8444	−	−
	Specificity (%)	0.8611	−	−
	PPV (%)	0.1461	−	−
	NPV (%)	0.9949	−	−
	AUC (95%CI) 95% CI *P* value	0.8834 0.8315–0.9354 0.0000	− − −	− − −
***M. abscessus* from PTB**	Cut-off value	<0.3565 IU/ml	<4 SFCs	<1 SFCs
	Sensitivity (%)	0.8999	0.7942	0.8452
	Specificity (%)	0.8058	0.7576	0.7879
	PPV (%)	0.3349	0.1645	0.1932
	NPV (%)	0.9867	0.9839	0.9883
	AUC (95%CI) 95% CI *P* value	0.8783 0.8417–0.9149 0.0000	0.8199 0.7410–0.8987 0.0000	0.8383 0.7690–0.9076 0.0000

−, not available.

## Discussion

Previous reports about the diagnostic performance of IGRAs in distinguishing NTM infections remain limited and their conclusions were very heterogeneous. Hermansen reported the QFT-G positivity rate was 8% (4/53) in definite NTM disease and 31% (15/49) in possible disease with colonization, while the overall rate of positive QFT-G in pulmonary NTM disease defined patients was 18% (81/462) by their systematic review (Hermansen et al., 2014). Augustynowicz-Kopeć reported that the positive IGRAs result was 8% in NTM definite patients (3/39) (Augustynowicz-Kopeć et al., 2019). Wang stated that the positivity rate of T-SPOT.TB was 53.4% (31/58) among the probable and definite NTM groups, 53.5% (15/28) for probable cases and 53.3% (16/30) for definite cases (Wang et al., 2016). Siegel reported the lowest positivity rate (2.0%, 1/51) for both QFT-G assay and a new-generation QuantiFERON-TB Gold Plus (QFT-Plus) assay in patients with MAC or *M. abscessus* (Siegel et al., 2018). In our article, with the traditional cut-off, positivity rate for QFT-G assay and T-SPOT.TB would be respectively 30.8% (317/1029) and 34.7% (181/522). The heterogeneity of these studies might be ascribed into several reasons as follow.

Firstly, the case number with valid IGRA results (all less than 100 from previous reports) limited the accuracy of analyses and comparison. Secondly, the lack of completed control group (PTB and other respiratory diseases) for comparisons may limit the estimation the discriminating power. Thirdly, these researches evaluated the discriminating power for NTM by calculating the positivity rate of IGRAs with cut-off values designed to differentiate TB, while distinguishing NTM, a modified cut-off should be optimized by ROC technique in the target population (Greiner et al., 2000).

In our research, we finally obtained 1029 valid results of QFT-G and 521 valid results of T-SPOT.TB among almost 80,000 patients enrolled from 2011 to 2019 with potential NTM disease, which was 10 times larger than the research by Andrejak (Andrejak et al., 2010) and 10 times larger than the research by Wang in China (Wang et al., 2016). Based on a large consecutive data set, we evaluated the discriminatory power of IGRAs between NTM and PTB with the area under the ROC curve (AUC), which is regarded as a global summary statistic of diagnostic accuracy (Greiner et al., 2000). Our data suggested a moderate accuracy with AUC > 0.7 for IGRAs to differentiate these diseases. Therefore, we recommended the application of IGRA tools (T-SPOT. TB and QFT-G) to distinguish NTM in AFB smear-positive patients who were composed of only NTM and PTB patients, and QFT-G may be preferred due to its higher AUC value.

Furthermore, our data showed that no significant difference of discrimination power was identified between NTM and Control patients by IGRAs. Since smear-negative patient group was composed of NTM, PTB, latent tuberculosis infection (LTBI) and other respiratory diseases, and the IGRA tools may only differentiate PTB and LTBI from this group, the remaining NTM and other respiratory diseases could not been further differentiated by this method. This result indicated the limitation of IGRAs in distinguishing NTM from AFB smear-negative patients Therefore, our study recommended the application of IGRAs to diagnose NTM in AFB smear-positive patients.

**Figure 1:**
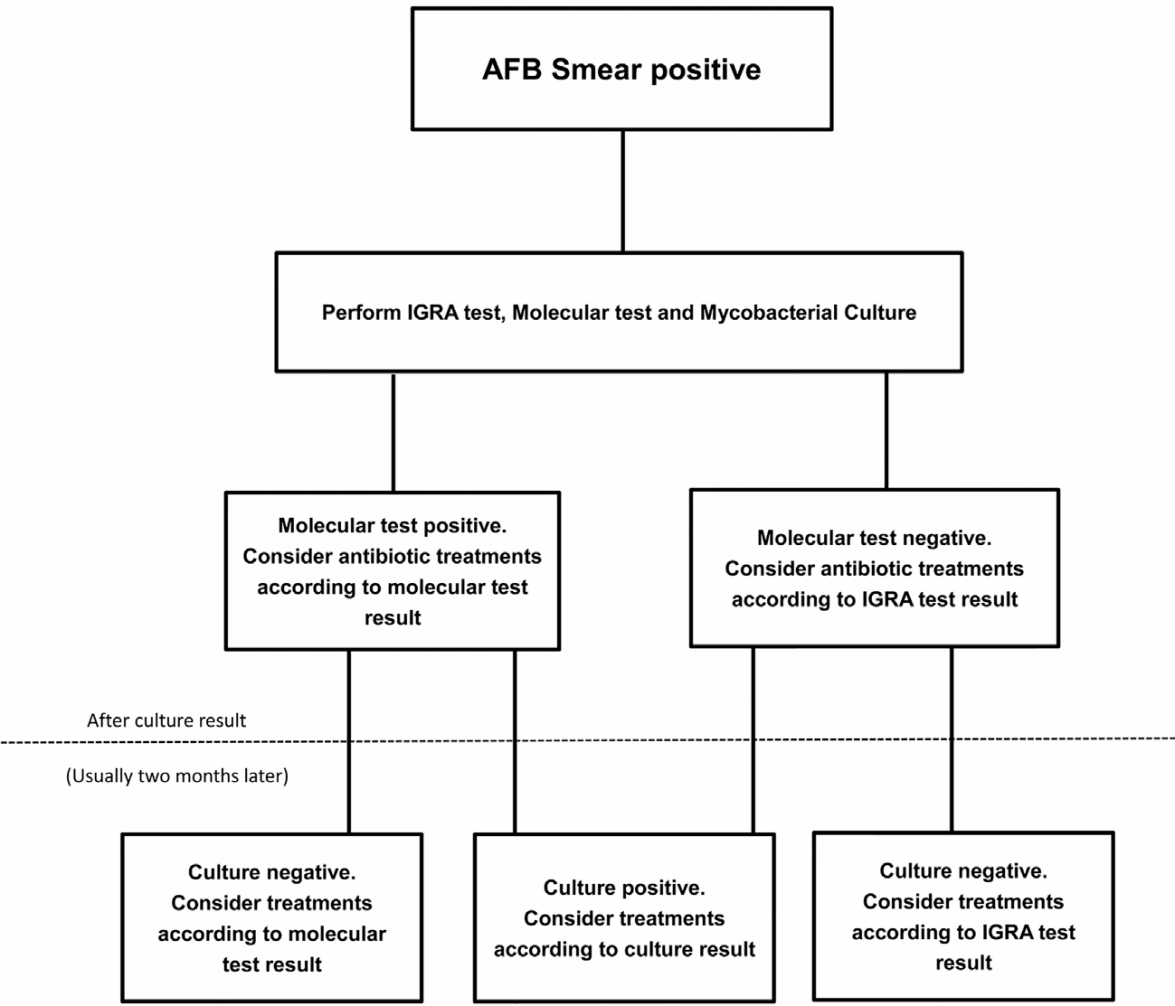
A suggested algorithm for the nontuberculous mycobacterial (NTM) investigation of smear positive individuals.

A number of methods detecting NTM from respiratory samples had been applicable, including culture and several molecular techniques. Culture usually provides the most reliable evidence for diagnosis, however, it is time-consuming and may provide negative results even in AFB smear-positive patients, making early diagnosis difficult (Haworth et al., 2017). The molecular techniques (PCR restriction analysis, Anyplex MTB/NTM detection assay, GenoType Mycobacteria Direct test) could be faster and highly specific but still share the limitation to be occasionally ineligible (Haworth et al., 2017). The PCR restriction analysis was reported to successfully amplify mycobacterial DNA in only 60% (72/121) of NTM patients with AFB smear-positive sputum Kim et al., 2008). Franco-Alvarez de Luna et al. reported that the GenoType Mycobacteria Direct test, which is capable of detecting TB and four atypical mycobacterium species, detected 92% (93/101) of tuberculosis patients and 22% (6/27) of nontuberculosis patients in culture positive samples (Franco-Alvarez De Luna et al., 2006). Perry et al. showed that the rate of negative results from a molecular tool (Anyplex MTB/NTM detection assay) was 11% (10/91) in smear positive patients (Perry et al., 2014). Kim et al. also reported two real time PCR assays (Anyplex plus MTB/NTM Detection kit and Genedia MTB/NTM Detection kit) had respectively 82%(14/17) and 76%(13/17) positive rate in NTM culture positive patients (Kim et al., 2020). Shin et al. reported the positive rate of Genedia MTB/NTM Detection kit was only 23%(16/69) in NTM culture positive patients (Shin et al., 2020). In our research, IGRA tools (QFT-G, ESAT-6 and CFP-10) could detect 87%, 79% and 76% NTM in smear-positive patients, and still could play an alternative or complementary role in discrimination of NTM, especially while negative test results occur with other tools. A suggested algorithm for the investigation of smear positive individuals is shown in **Figure 1**.

Previous studies usually adopted the recommended cut off value of IGRAs which were designed for differentiating PTB from Controls, and some researchers already revealed that a revised cut-off value may increase the sensitivity to detect NTM disease. Kobashi et al. reported when the cut-off value of positive response for QFT-G changed from 0.35 to 0.20 IU/ml, the sensitivity to detect NTM disease (*M. kansasii* disease) increased from 52% to 82%, while specificity decreased from 93% to 91% (Kobashi et al., 2009). Similarly, our survey showed that modified cut-off values for discriminating NTM (ESAT-6 < 4 SFCs; CFP-10 < 3 SFCs and QFT-G < 0.667 IU/mL) could also improve their summary accuracies. For QFT-G, its sensitivity would decrease from 90% to 87%, while specificity would increase from 69% to 75%. For T-SPOT, its sensitivities for ESAT-6 and CFP-10 would grow from 70% and 66% to 79% and 76%, respectively; correspondingly their specificities would decrease from 71% and 77% to 63% and 68%.

Meanwhile, some NTM species (*M. kansasii*, *M. marinum* and *M. szulgai*) sharing RD1 with *M. tuberculosis* showed similar positive results with IGRAs as *M. tuberculosis* (Hermansen et al., 2014; Chen et al., 2018; Augustynowicz-Kopeć et al., 2019), and the feasibility of differentiating these species from PTB was doubtful. Our analysis suggested there was no statistically significant difference of IGRAs performances between any species of NTM. Additionally, the ROC curve in this study showed that QFT-G displayed a moderate accuracy (AUC = 0.8834) when distinguishing some RD1-posesssing NTM species (at least *M. kansasii*), which was inconsistent with other researchers and might be caused by a revised cut-off (< 0.8180 IU/mL) to amplify the difference between these RD1-posesssing NTM species patients and PTB patients.

Last, we need to emphasize the limitations of our work. First, the patients were collected from a single center in a high TB incidence country, which may limit its generalizability in low TB incidence country. More intercontinental surveys are needed to expand its universality. Second, as a retrospective review, bacterial species identification could not be performed in all the patients. Therefore, the analysis of discriminatory power was unavailable for species with scarce isolations, including *M. gastri*, *M. marinum*, *M. szulgai* and so on. Third, due to the retrospective nature of this article, the BCG vaccination statuses of our patients are unavailable. BCG vaccination was regarded to be associated with durable IFN-g responses and its impact on the performances of IGRA tools should not be neglected.

To our best knowledge, this study is one of the largest assessing IGRAs with valid results in discriminating NTM infections from both AFB smear positive (PTB) and AFB smear negative patients. T-SPOT.TB and QFT-G were performed in patients with NTM infection, PTB and other respiratory diseases. Our results revealed that, with modified cut-off values, these IGRAs possessed the potential in differentiating NTM disease from PTB disease in AFB smear-positive patients. Furthermore, for some species of NTM (MAC, *M. abscessus*, even RD1 possessing mycobacteria), the T-SPOT.TB or QFT-G had moderate discriminatory power. However, since many respiratory diseases (pneumonia et al.) in AFB smear-negative patients share similar IGRAs results with NTM, the discrimination power of IGRA tools for NTM in AFB smear-negative patients may be limited in diagnosis. In conclusion, our study provided new insights into the diagnostic performance of IGRAs in differentiation of NTM infection and PTB, and provided more guidance to promote the diagnostic accuracy of PTB and NTM infection in the clinic.

## Data Availability Statement

The raw data supporting the conclusions of this article will be made available by the authors, without undue reservation.

## Ethics Statement

The studies involving human participants were reviewed and approved by The Institutional Review Board of Shanghai Pulmonary Hospital affiliated with Tongji University. Written informed consent from the participants was not required to participate in this study in accordance with the national legislation and the institutional requirements

## Author Contributions

HC, CY, HX, and WS contributed to study design. CY, XL, LF, HX, and WX performed the experiments. CY and XL performed data analysis. CY and HC wrote the manuscript. All authors contributed to the article and approved the submitted version.

## Funding

This work is supported by Shanghai top priority of clinical medical center and key discipline construction plan (2017ZZ02003), Shanghai science and technology commission project (20Y11901500) and Shanghai clinical research center for infectious disease (tuberculosis, 19MC1910800).

## Conflict of Interest

The authors declare that the research was conducted in the absence of any commercial or financial relationships that could be construed as a potential conflict of interest.
