# Supplementary material for: Performance of Interferon-Gamma Release Assays in the Diagnosis of Nontuberculous Mycobacterial Diseases—A Retrospective Survey From 2011 to 2019
Source: Front Cell Infect Microbiol. 2021 Feb 18;10:571230. doi: 10.3389/fcimb.2020.571230 (PMC7930076; doi:10.3389/fcimb.2020.571230)
Supplement: Supplementary file 1 [file DataSheet_1.docx]

**Supplementary files for:**

**Performance of interferon-gamma release assays in the diagnosis of nontuberculous mycobacterial diseases - a retrospective survey in China from 2011-2019**

Chi Yang, Xuejiao Luo, Lin Fan, Wei Sha, Heping Xiao, Haiyan Cui^*^

Shanghai clinical research center for tuberculosis, Shanghai Pulmonary Hospital, Tongji University School of Medicine, Shanghai 200433, People’s Republic of China

* Address correspondence to Haiyan Cui, Tel: +86 21 65115006, email: [cuihaiyan@tongji.edu.cn](mailto:cuihaiyan@tongji.edu.cn)

**Table S1 The quantitative results of IGRAs for NTM, NTM species, PTB and Controls.**

| Quantitative results | ***N*TM** | **There major species NTMs** | | | **PTB** | **Controls** |
| --- | --- | --- | --- | --- | --- | --- |
|  | *N* = 1407 | *M. kansasii*  *N* =36 | *M. abscessus*  *N* =151 | MAC  *N* =232 | *N* = 1828 | *N* = 2652 |
| **ESAT-6**  (SFCs) | 6.93±0.73  *n* = 521 | *−* | 5.76±3.37  *n* = 33 | 3.20±1.03  *n* = 41 | 17.00±0.77  *n* = 549 | 3.83±0.24  *n* = 1288 |
| **CFP-10** (SFCs) | 6.66±0.80  *n* = 521 | *−* | 4.45±3.052  *n* = 33 | 3.80±1.50  *n* = 41 | 23.51±1.33  *n* = 549 | 3.96±0.31  *n* = 1288 |
| **QFT-G**  (IU/ml) | 1.31±0.09  *n* = 1029 | 0.82±0.34  *n* =36 | 1.12±0.24  *n* = 139 | 1.11±0.19  *n* = 211 | 5.88±0.11  *n* = 1279 | 1.46±0.08  *n* = 1364 |

Data presented as mean ± SD. *N* represented for enrolled patient number, *n* represented for patient number with valid data from IGRAs. −: not available.

**Table S2. The IGRAs quantitative results comparisons between pairs from NTM, PTB, Controls and species of NTM.**

| **IGRAs** |  | ***M.kansasii*** | ***M. abscessus*** | **MAC** | **NTM** |
| --- | --- | --- | --- | --- | --- |
| **ESAT-6** | **PTB** | − | ***P* = 0.0025*** | ***P* = 0.0000*** | ***P* = 0.0000*** |
|  | **Controls** | − | *P* = 0.5717 | *P* = 0.6396 | ***P* = 0.0000*** |
|  | ***M. abscessus*** | − | − | *P* = 0.4305 | *P* = 0.6982 |
|  | **MAC** | − | *P* = 0.4305 | − | *P* = 0.1554 |
| **CFP-10** | **PTB** | − | ***P* = 0.0000*** | ***P* = 0.0000*** | ***P* = 0.0000*** |
|  | **Controls** | − | *P* =0.8031 | *P* = 0.9280 | ***P* = 0.0017*** |
|  | ***M. abscessus*** | − | − | *P* = 0.8397 | *P* = 0.5001 |
|  | **MAC** | − | *P* = 0.8397 | − | *P* = 0.3221 |
| **QFT-G** | **PTB** | ***P* = 0.000*** | ***P* = 0.000*** | ***P* = 0.000*** | ***P* = 0.000*** |
|  | **Controls** | *P* = 0.069 | *P* = 0.1810 | *P* = 0.0890 | *P* = 0.1853 |
|  | ***M.kansasii*** | − | *P* = 0.5549 | *P* = 0.5389 | *P* =0.1676 |
|  | ***M. abscessus*** | *P* = 0.5549 | − | *P* = 0.9912 | *P* =0.4547 |
|  | **MAC** | *P* = 0.5389 | *P* = 0.9912 | − | *P* =0.3606 |

*Statistically significance (*P* < 0.0001) are shown in bold.

−: not available


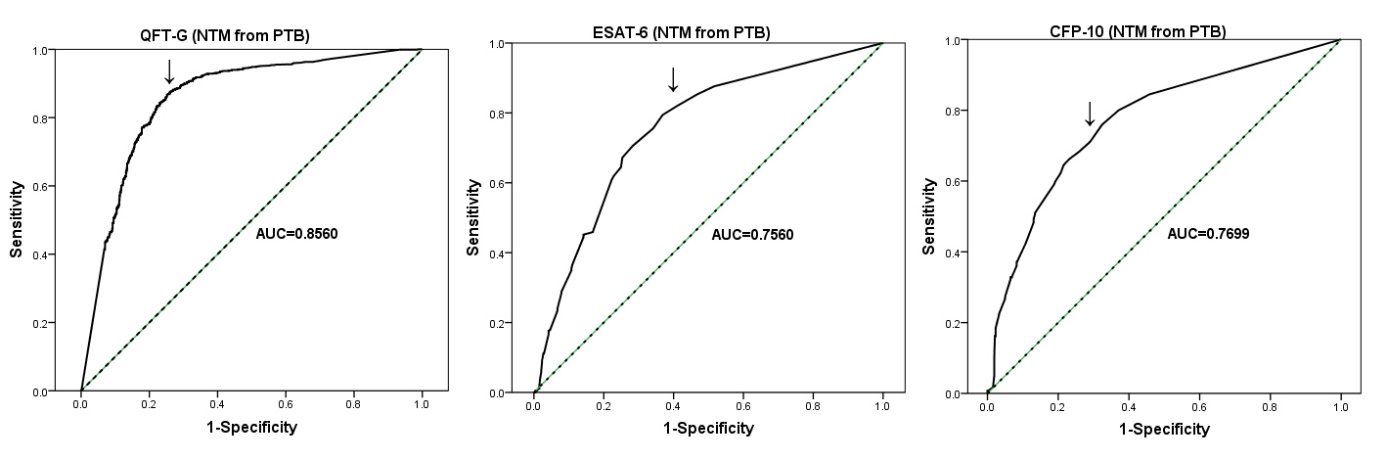


**Figure S1**. Receiver Operating Characteristic (ROC) curves for ESAT-6, CFP-10 and QFT-G while discriminating NTM from PTB. The dotted diagonal line indicates no discrimination. The optimal cut-off point in ROC is marked with arrow.


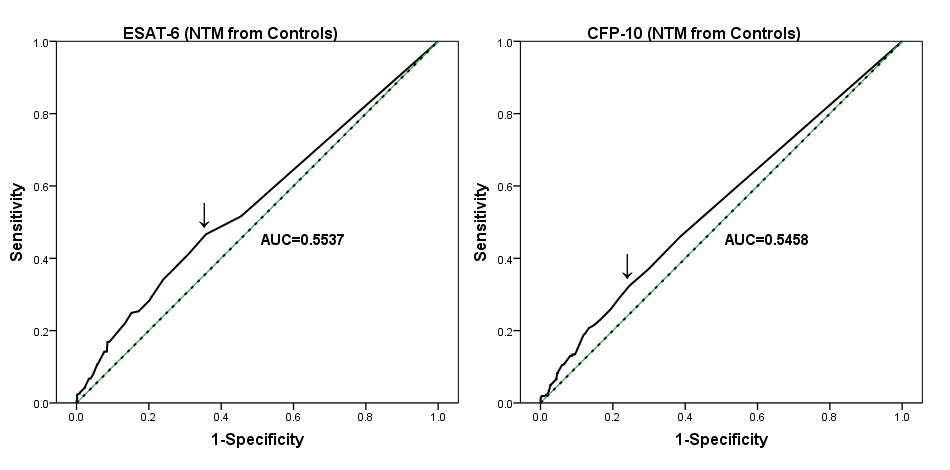


**Figure S2**. Receiver Operating Characteristic (ROC) curves for ESAT-6 and CFP-10 while discriminating NTM from Controls. The dotted diagonal line indicates no discrimination. The optimal cut-off point in ROC is marked with arrow.


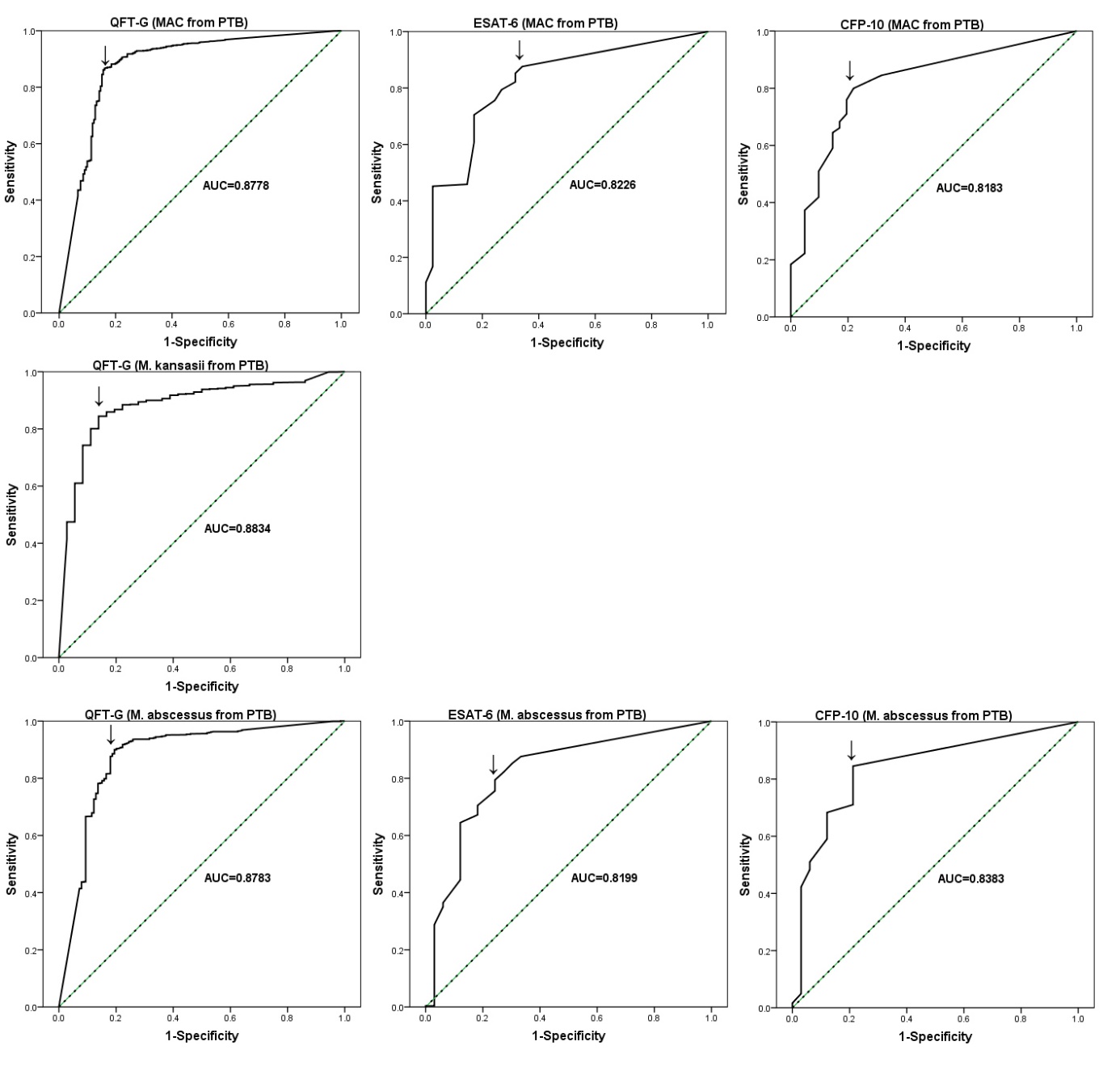


**Figure S3**. Receiver Operating Characteristic (ROC) curves for ESAT-6, CFP-10 and QFT-G while discriminating species of NTM from PTB. The dotted diagonal line indicates no discrimination. The optimal cut-off point in ROC is marked with arrow.
